# Development and Validation of LC-MS/MS Method for Nintedanib and BIBF 1202 Monitoring in Plasma of Patients with Progressive Pulmonary Fibrosis Associated with Systemic Sclerosis

**DOI:** 10.3390/pharmaceutics17121553

**Published:** 2025-12-02

**Authors:** Anna Kiełczyńska, Edyta Gilant, Tomasz Pawiński, Iwona Szlaska, Katarzyna Buś-Kwaśnik, Edyta Pesta, Daria Kuc, Brygida Kwiatkowska

**Affiliations:** 1 Department of Drug Chemistry, Pharmaceutical and Biomedical Analysis, Medical University of Warsaw, Banacha 1, 02-097 Warsaw, Poland; anna.a.kielczynska@gmail.com (A.K.); iwona.szlaska@wum.edu.pl (I.S.); 2 Łukasiewicz Research Network—Industrial Chemistry Institute, Rydygiera 8, 01-793 Warsaw, Poland; edyta.gilant@ichp.lukasiewicz.gov.pl (E.G.); katarzyna.bus-kwasnik@ichp.lukasiewicz.gov.pl (K.B.-K.); edyta.pesta@ichp.lukasiewicz.gov.pl (E.P.); 3 Clinic of Early Arthritis, National Geriatrics, Rheumatology and Rehabilitation Institute, Spartańska 1, 02-637 Warsaw, Poland; daria.kuc@spartanska.pl (D.K.); brygida.kwiatkowska@spartanska.pl (B.K.)

**Keywords:** nintedanib, BIBF 1202, LC-MS/MS, therapeutic drug monitoring, progressive pulmonary fibrosis, systemic sclerosis

## Abstract

**Background**: Nintedanib (NIN), an intracellular inhibitor of tyrosine kinases that inhibits processes fundamental to the progression of pulmonary fibrosis (PPF), is used in the treatment of patients with PPF associated with systemic sclerosis. During NIN therapy, adverse events lead to a permanent dose reduction and treatment discontinuation. Therapeutic drug monitoring (TDM) can be used to manage and optimize drug administration based on the measurement of drug concentrations. Therefore, TDM can be helpful in minimizing the impact of adverse events and help patients remain in therapy. The aim of this study was to develop and validate a new bioanalytical UPLC-MS/MS method enabling the determination of NIN and its active metabolite in the plasma of patients with PPF associated with systemic sclerosis. **Methods**: Sample preparation was carried out using protein precipitation with an extraction mixture: acetonitrile neutralized with 2 M sodium carbonate. Analytes and the internal standard (intedanib-d3) were monitored using mass spectrometry (MS) and positive-ion-mode electrospray ionization by MRM. Chromatographic analysis was performed on a Zorbax SB-C18 column kept at 40 °C using isocratic elution. The mobile phase contained 0.1% formic acid in water; acetonitrile (35:65 *v*/*v*) was pumped at a flow rate of 0.3 mL/min. The analysis time was 5 min. **Results**: The method was verified according to the EMA guidelines over a concentration range of 2.00–200.00 ng/mL. The correlation coefficients for the calibration curves were found to be 0.9991 and 0.9957 for NIN and its metabolite BIBF 1202, respectively. The within- and between-run precision and accuracy of LLOQ were evaluated for NIN and BIBF 1202 to be within RSD 2.96%, 4.53%, 5.51%, and 6.72% and in the ranges of 102.2–107.3%, 98.0–101.8%, 104.3–114.2%, and 99.1–104.9, respectively. The stability of the analytes in plasma after 4 h at 30 °C was found to be satisfactory, meeting the assumed bias criteria below 15%. **Conclusions**: The proposed method was successfully applied to analyze two active compounds—NIN and BIBF 1202—in plasma samples at two time points: trough (pre-dose concentration) and 2–3 h (maximum concentration) after the administration of NIN.

## 1. Introduction

Nintedanib (NIN), a tyrosine kinase inhibitor, has been used in the treatment of progressive pulmonary fibrosis associated with systemic sclerosis over the last 10 years. Systemic sclerosis is a rare and heterogeneous autoimmune disease characterized by immune dysregulation, microvascular damage, and organ fibrosis. In patients with progressive pulmonary fibrosis, treatment with NIN (150 mg twice daily) showed disease progression by reducing the rate of decline of the forced vital capacity (FVC). NIN has shown antifibrotic, anti-inflammatory, and vascular-remodeling effects in several animal models demonstrating aspects of systemic sclerosis [1,2,3,4]. Due to the high inter-individual pharmacokinetic variability of NIN, which can impact both the efficacy and safety of therapy, therapeutic drug monitoring (TDM) is required. Therapeutic drug monitoring is an essential element of personalized pharmacotherapy, allowing for treatment optimization by taking into account differences in drug pharmacokinetics and pharmacodynamic effects between patients. The primary goal of TDM is to increase treatment efficacy while minimizing the risk of adverse effects and toxicity. For TDM to be clinically justified, specific pharmacological and clinical criteria must be met. The most important of these are a narrow therapeutic-concentration range, wide variation in the pharmacokinetic profile, an established relationship between concentration and clinical effect or toxicity, and the ability to accurately and reproducibly determine drug concentration. Small molecule kinase inhibitors have a narrow therapeutic window, which may consequently cause an increase in NIN concentration above the upper limit of the therapeutic index, leading to toxicity, where ineffective treatment occurs when the target concentration is not reached [5,6,7]. The most frequent adverse effect during NIN therapy was diarrhea, which occurred at a rate of 60.1% in patients who continued NIN and 71% in patients who initiated NIN [8]. For this purpose, a sensitive, selective, and rapid method of determining NIN was developed. In addition, the method allows for the simultaneous determination of the active metabolite of NIN. The presented method was ultimately successfully implemented in the analysis of biological samples taken from patients, which confirms its usefulness in real clinical conditions.

The LC-MS/MS method for NIN and its metabolite BIBF 1202 was developed to obtain accurate and precise results with the sample preparation procedure, which is simple and consists of new logical steps that can be easily performed in any laboratory. BIBF 1202, as the main metabolite, is formed as a result of rapid hydrolytic ester cleavage of the methyl ester moiety. According to our knowledge, in the described method is presented, for the first time, the simultaneous determination of NIN and its metabolite in the plasma of patients using real-world evidence. There are a few procedures found in the literature regarding the development and validation of the LC-MS/MS method for the determination of NIN, but among them, only two are dedicated to analyzing NIN in the human plasma of patients with idiopathic and progressive fibrosis [9,10,11,12]. Also, it was found that a few articles were published that included a quantity analysis of rat biological fluids [13,14,15].

Comparing the literature’s methods with those presented above, we can note that we developed a sensitive LC-MS/MS assay that enables the simultaneous quantification of nintedanib and BIBF 1202 with proper accuracy and precision according to the European Medicines Agency guidelines. This study reports that the LC-MS/MS method enables the determination of two concentrations at the trough level and 2 h after morning dose administration, the absorption stage of NIN. The method validation was conducted based on the newest EMA guidelines [16].

## 2. Materials and Methods

### 2.1. Chemical Reagents

Nintedanib (NIN) and BIBF 1202 reference standards were obtained from MedChemExpress (Monmouth Junction, NJ, USA). The internal standard (IS), intedanib-d3 (NIN-d3), was purchased from Toronto Research Chemicals (Toronto, ON, Canada). Methanol, acetonitrile (ACN), isopropanol, and dimethyl sulfoxide (DMSO) were supplied by Chemsolve (Łódź, Poland). Formic acid was purchased from Avantor Performance Materials Poland (Gliwice, Poland). Sodium carbonate was obtained from Sigma-Aldrich (Merck Group, Laramie, WY, USA). Purified water was produced using a Milli-Q purification system (Merck Millipore, Darmstadt, Germany).

**Figure 1:**
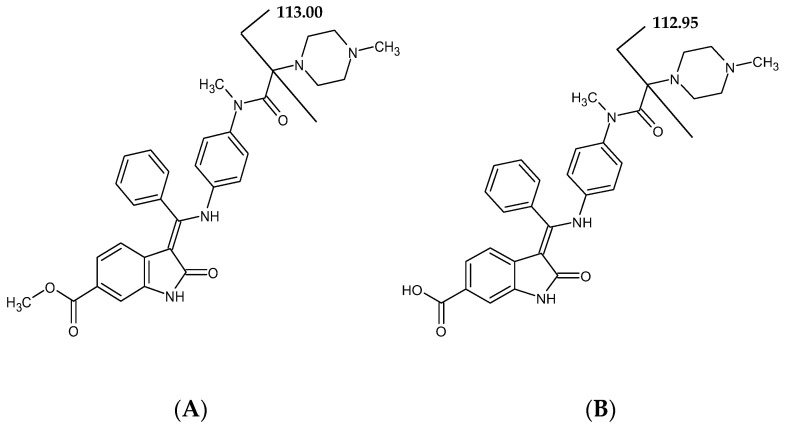
Structures of analyzed compounds: nintedanib (NIN) (**A**), BIBF 1202 (**B**) with fragmentations.

The chemical structures of NIN and BIBF 1202 are presented in Figure 1.

### 2.2. Stock and Working Solutions

Stock solutions were prepared by dissolving the reference standards (NIN, BIBF 1202, and intedanib-d3) in DMSO to a concentration of 0.20 mg/mL. Solutions of NIN and BIBF 1202 were subsequently combined. Primary working solutions containing a mixture of NIN and BIBF 1202, as well as NIN-d3, were prepared by diluting the stock solutions in 80% acetonitrile. Lower-concentration working solutions were obtained by further diluting the primary stock solutions in 80% ACN. Ultimately, ten working solutions were prepared for the NIN and BIBF 1202 mixture, and two for intedanib-d3, at the following concentrations: 20.00, 50.00, 200.0, 600.00, 1000.00, 1400.00, 1700.00, 2000.00, 3400.00, and 10,000.00 ng/mL for the mixture and 10.00 and 1.00 ng/mL for NIN-d3.

### 2.3. Calibration Standards (CS) and Quality Control Samples (QC)

For the preparation of calibration standards, 180 µL of blank human plasma was spiked with 20 µL of the appropriate working solution. Calibration standards were prepared at the following nominal concentrations: 2.00, 5.00, 20.00, 60.00, 100.00, 140.00, 170.00, and 200.00 ng/mL. QC samples were prepared in the same manner at concentrations of 5.00, 20.00, 100.00, 170.00, and 340.00 ng/mL. Both CS and QC samples were made immediately prior to protein precipitation.

### 2.4. Patient Samples and Protocol of Sampling

Whole blood was collected via standard venipuncture from 24 patients diagnosed with CTD-PPF. Patients received treatment with NIN, of whom 8 were administered a dose of 100 mg twice daily and 16 received 150 mg twice daily. Blood samples were obtained prior to the first daily NIN dose (trough concentration, C_0_) and 2–3 h afterward (maximum concentration, C_max_). Following collection, samples of whole blood with NIN and metabolite were centrifuged, and then plasma was stored at −70 °C. This study was approved by the Local Bioethics Committee of the National Institute of Geriatrics, Rheumatology, and Rehabilitation in Warsaw (KBT-5/3/2024).

### 2.5. Sample Preparation

**Figure 2:**
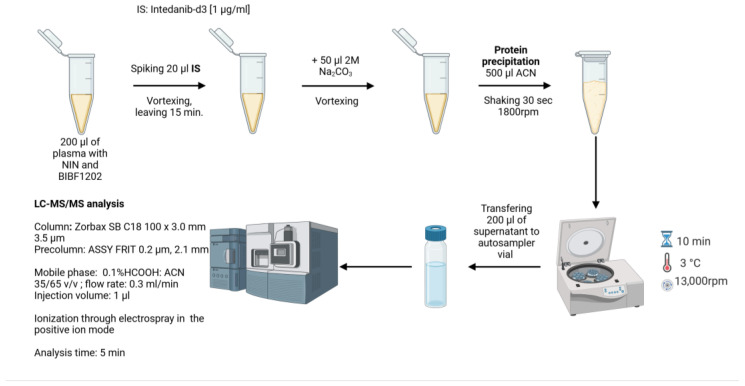
Sample preparation and analysis.

To prepare the samples, 200 μL of plasma was spiked with 20 μL of IS (NIN-d3) and vortexed. Protein precipitation was carried out using 500 μL of chilled ACN solution after we previously neutralized the samples with 50 μL of 2 M sodium carbonate (Na_2_CO_3_). The samples were subsequently mixed on an orbital shaker (Vibrax, IKA, Staufen, Germany) for 30 s at 1800 and centrifuged for 10 min at 13,000 rpm, 3 °C. Then, 200 μL of the supernatant was transferred into autosampler vials for LC-MS/MS analysis under the conditions described in Section 2.6 and Section 2.7. The sample preparation scheme for analysis is shown in Figure 2.

### 2.6. Chromatographic Equipment and Conditions

The UPLC Acquity I-class Plus system (Milford, MA, USA, Waters), equipped with a quaternary gradient pump, column oven, degasser unit, and autosampler, was used. The autosampler and column were maintained at 10 ± 2 °C and 40 ± 2 °C, respectively. The isocratic elution was carried out using a mobile phase consisting of 0.1% formic acid in water and acetonitrile (35:65, *v*/*v*). The mobile phase flowed through a Zorbax SB-C18 column (100 mm × 3.0 mm, 3.5 µm; Santa Clara, CA, USA, Agilent Technologies) at a flow rate of 0.3 mL/min. The system was equipped with an ASSY FRIT pre-column (0.2 µm, 2.1 mm; Milford, MA, USA, Waters). To prevent the carry-over effect, after the analysis, the injection needle was washed with a mixture consisting of acetonitrile, methanol, isopropyl alcohol, water, and formic acid (250:250:250:250:1, *v*/*v*/*v*/*v*/*v*).

### 2.7. MS Equipment and Conditions

Mass spectrometric analysis was performed using a XEVO-TQ-XS quadrupole mass spectrometer (Milford, MA, USA, Waters). Multiple Reaction Monitoring (MRM) chromatograms were acquired and processed with MassLynx 4.2 software (Milford, MA, USA, Waters). Ionization was carried out in positive ion mode using electrospray ionization (ESI+). MS parameters of instruments and analyzed compounds are detailed in Table 1.

### 2.8. Method Validation

The bioanalytical method validation of NIN and BIBF 1202 determination in human plasma of patients with progressive pulmonary fibrosis associated with systemic sclerosis was performed according to the European Medicines Agency (EMA) guidelines. The parameters confirming the robustness of the obtained analytical results have been defined as linearity, lower limit of quantification (LLOQ), accuracy, precision, carry-over, selectivity, matrix effect, recovery, and stability of NIN and BIBF 1202 in human plasma.

#### 2.8.1. Linearity

The linearity of the developed method for determining NIN and BIBF 1202 was assessed to be in the range of 2.00–200.00 ng/mL for both compounds. Six calibration curves were prepared, where eight calibration standards (at the following concentrations: 2.00, 5.00, 20.00, 60.00, 100.00, 140.00, 170.00, and 200.00 ng/mL), along with a blank sample (without additional analytes or IS) and a zero sample (plasma with the addition of IS), were studied. Linearity was assessed using linear regression, where the coefficient of determination (R^2^) should be ≥0.95. The weighting was selected according to the minimum sum of percentage relative errors (RE%). Accuracy of at least six out of eight non-zero calibration standards should fall within 85–115%, with the exception of LLOQ, for which 80–120% limits are allowed.

#### 2.8.2. Precision and Accuracy

Accuracy and precision were evaluated using LLOQ (2.00 ng/mL), and quality control samples (QC) were prepared at four concentrations—5.00, 20.00, 100.00, and 170.00 ng/mL—defined respectively as LQC1, LQC2, MQC, and HQC. Each QC sample was analyzed using at least five replicates within a single run (within-run) and in three independent runs (between-run) over at least two days. For each QC concentration level, the accuracy must be within 85–115% (for LLOQ, 80–120%), and the precision (CV%) must be ≤15% (for LLOQ, 20%).

#### 2.8.3. Carry-Over Effect

Carry-over was evaluated by injecting a blank sample (BS) immediately after the sample in which the concentration of NIN and BIBF 1202 reached the highest calibration standard (200.00 ng/mL, ULOQ). The parameter was also examined for IS. The procedure was repeated six times. The signal obtained in the blank sample must not exceed 20% of the analyte’s lower limit of quantification (LLOQ) or 5% of the internal standard (IS) response.

#### 2.8.4. Matrix Effect

Matrix effects (MEs) for both NIN and BIBF 1202 were evaluated using human plasma from six sources, including hemolyzed and lipemic matrices. The parameter was tested for quality samples of NIN and BIBF 1202 at the following levels of two concentrations: LQC_1_ (5.00 ng/mL) and HQC (170.00 ng/mL). Each was measured in six replicates.

#### 2.8.5. Recovery

To determine the recovery, two sets of results were compared, obtained from samples where equal amounts of NIN and BIBF 1202 were added to the plasma before and after the precipitation procedure. The recovery test was conducted for the QC samples at concentrations of 5.00, 100.00, and 170.00 ng/mL (LQC_1_, MQC, and HQC, respectively), including IS; all samples were examined six times. The extent of recovery of an analyte and of the IS should be consistent. The recovery of the internal standard (IS) should be independent of the concentration of the analytes.

#### 2.8.6. Selectivity

The selectivity was evaluated for samples of blank plasma (without addition analytes or IS) obtained from eight different sources, including hemolyzed and hyperlipidemic plasma. The interferences with the retention times of NIN, BIBF 1202, and IS must not exceed 20% of the LLOQ for the analytes or 5% for the IS.

#### 2.8.7. Stability

The stability of NIN and BIBF 1202 in human plasma, including in the short term, autosampler, and freeze–thaw, was evaluated for the QC samples analyzed at two concentrations, 5.00 ng/mL (LQC1) and 170.00 ng/mL (HQC), studied in six replicates per test. The short-term stability of NIN and BIBF 1202 was assessed for the samples that were stored at ambient temperature (≤30 °C) for at least two hours, which were analyzed and compared with freshly prepared samples. Autosampler stability was determined by storing samples in the autosampler at 10 ± 2 °C for at least 18 h. Freeze–thaw stability was examined over three cycles, in which samples were frozen at ≤−14 °C for 12 h and subsequently thawed at ambient temperature (≤30 °C).

#### 2.8.8. Dilution Integrity

The integrity test of the dilution was conducted using control plasma solutions with a final concentration of 340.00 ng/mL for both NIN and BIBF 1202. The samples were diluted in a 1:1 (*v*/*v*) ratio using control plasma. The obtained concentration values were multiplied by the dilution factor (2×). The test was performed in six repetitions. It has been shown that the dilution of samples had no effect on the accuracy or precision of the measured concentration of the analytes.

#### 2.8.9. Reinjection Reproducibility

The reinjection reproducibility was assessed using a minimum of five replicates of injection from one vial at the four concentrations—5.00, 20.00, 100.00, and 170.00 ng/mL—for both compounds NIN and BIBF 1202. At each examined concentration level, the accuracy should be in the range of 85–115%, and the precision must remain within ±15% of the nominal value.

#### 2.8.10. Incurred Sample Reanalysis

ISR (incurred sample reanalysis) is used to assess the reliability of a bioanalytical method. The test is performed by repeating the analysis of selected study samples in separate sequences on different days using the same bioanalytical method. The percentage difference between the repeated result and original result should be calculated based on their mean value to confirm the reproducibility of the bioanalytical method, and the percentage difference should be within ±20% for at least 67% of the reanalyzed study samples.

## 3. Results

This section is divided by subheadings. It should provide a concise and precise description of the experimental results, their interpretation, and the experimental conclusions that can be drawn.

### 3.1. Method Development

**Figure 3:**
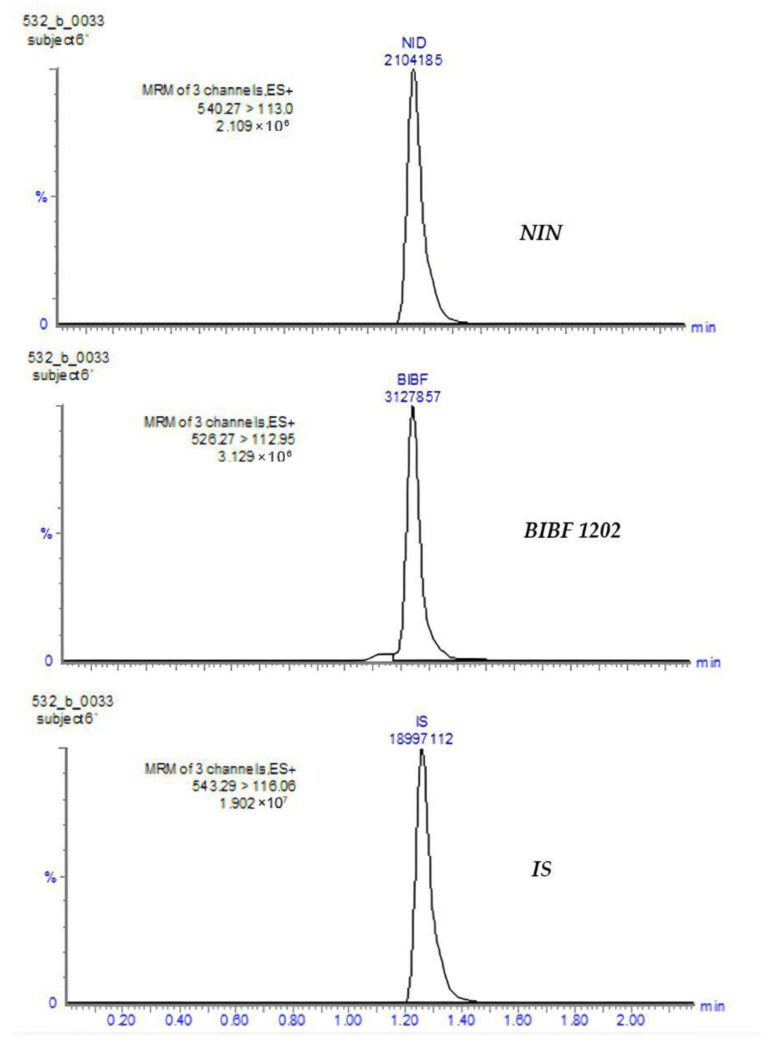
Chromatograms of NIN, BIBF 1202, and IS in the patient sample after administration of 100 mg dose at concentrations of 12.3 ng/mL NIN and 28.0 ng/mL BIBF 1202.

The determination of NIN and its metabolite BIBF 1202 in human plasma was performed using UPLC-MS/MS equipment. Optimization of the chromatographic conditions, including the selection of the appropriate column and mobile phase composition, was carried out to adjust the analysis time, peak shape quality, and, most importantly, the ability to separate analyte signals. Optimal results were achieved by using a Zorbax SB-C18 column (100 mm × 3.0 mm, 3.5 µm; Agilent Technologies) as a stationary phase and a mobile phase consisting of 0.1% formic acid and acetonitrile (35:65, *v*/*v*). The flow rate was set to 0.3 mL/min, and separation was conducted under isocratic elution. The total chromatographic run time was 5 min and the retention time was 1.26 min for each analyte. Sample chromatograms are shown in Figure 3.

#### 3.1.1. Linearity

The calibration curve was constructed by plotting the analytes/IS peak area ratios against the nominal concentrations of the analytes. The calibration curves for the method of determining NIN and BIBF 1202 were calculated by a linear regression analysis with a weighting factor of 1/x^2^ for NIN and 1/y^2^ for BIBF 1202. Weighting factors were chosen according to the minimum sum of the percentage relative errors (RE%). The calibration curves were linear in the concentration range of 2.00–200.00 ng/mL for both analyzed compounds. The obtained values of the coefficient of determination (R^2^) were satisfactory and amounted to 0.9991 and 0.9957 for the NIN and BIBF 1202 analyses, respectively. The values of the regression parameters for the curve, described by the equation y = ax + b, were as follows: the slope (a) was 0.0086 for NIN and 0.0034 for BIBF 1202; and the intercepts (b) were 0.0026 and 0.0010 for NIN and BIBF 1202, respectively.

#### 3.1.2. Precision and Accuracy

The study of accuracy and precision for the within-run (one sequence) and between-run (three sequence) analyses for the quality control samples (QC) was conducted at five concentration levels corresponding to LLOQ, LQC1, LQC2, MQC, and HQC. The data regarding the assessment of both within-run and between-run accuracy and precision were in accordance with the EMA guidelines. For each studied QC concentration, the accuracy was within the acceptance criteria range of 85–115%, and the precision was below the limit of 15%. The LLOQ for both quantified compounds showed acceptable accuracy (in the range of 80–120%) and precision (not exceeding 20%) at a concentration of 2.00 ng/mL.

The results for accuracy and precision are presented in Table 2.

#### 3.1.3. Carry-Over Effect

We examined the peak area of the analyzed compounds on the chromatograms, where six blank samples were injected directly after the ULOQ samples. No signals exceeded 20% of the LLOQ or 5% of the IS at working concentration. The obtained data is in accordance with the EMA recommendations.

#### 3.1.4. Matrix Effect

The accuracy and precision for all six matrices studied, obtained from various sources, met the acceptance criteria, falling within the range of 85–115% for accuracy and not exceeding ≤15% for precision. The results of this study on the matrix effect on the determination of NIN and BIBF 12020 are presented in Table 3.

#### 3.1.5. Recovery

The recoveries of the analyzed compounds after protein precipitation as reproducible at all concentration levels were within the range of 83.73–87.27% for NIN and 71.65–72.64% for BIBF 1202. The recovery of the internal standard (IS) was independent of the concentration of the analytes. Details are provided in Table 4.

#### 3.1.6. Selectivity

Plasma sample chromatograms from eight different sources, including hemolyzed and hyperlipidemic plasma, showed interference in the retention times of NIN, BIBF 1202, and IS less than 20% of the LLOQ and 5% of the IS signals.

#### 3.1.7. Stability

Storing the plasma samples for 4 h at a temperature of ≤30 °C had no effect on NIN concentrations, but this duration resulted in a reduction in the BIBF signal. Keeping compounds for 2 h at ambient temperature (≤30 °C) did not cause changes in the concentrations of either NIN or BIBF 1202. Autosampler stability was examined by storing samples at 10 ± 2 °C for up to 48 h. No impact of three freeze–thaw cycles (freezing at ≤−14 °C and thawing at ambient temperature) was observed on the stability of NIN or BIBF 1202. Table 5 shows the results of the sample stability of the NIN and BIBF 1202 concentrations in different conditions.

#### 3.1.8. Dilution Integrity

The mean accuracy measured during the integrity test for NIN and BIBF 1202 (concentration 340.00 ng/mL, *n* = 6) was 108.7 and 102.6%, respectively; the precision was 2.66 and 2.13% for NIN and its metabolite, respectively.

#### 3.1.9. Reinjection Reproducibility

In the study of the reproducibility of reinjection, in which QC samples were measured at concentrations corresponding to LQC_1_, LQC_2_, MQC, and HQC, the accuracy results ranged from 91.4 to 107.9% for NIN and from 100.0 to 114.3% for BIBF 1202. The precision did not exceed 15% and reached a maximum of 1.66% for NIN and 1.33% for its metabolite. Accuracy and precision met the EMA acceptance criteria.

#### 3.1.10. Incurred Sample Reanalysis

Five samples for incurred sample reanalysis (ISR) were selected from all the samples from the patients. In the developed method of determination of NIN and BIBF 1202, the percentage difference between the original values and the reanalyzed values for both the compounds remained within the limits of ±20%. The results are presented in Table 6.

### 3.2. Patient Samples and Clinical Applications

**Figure 4:**
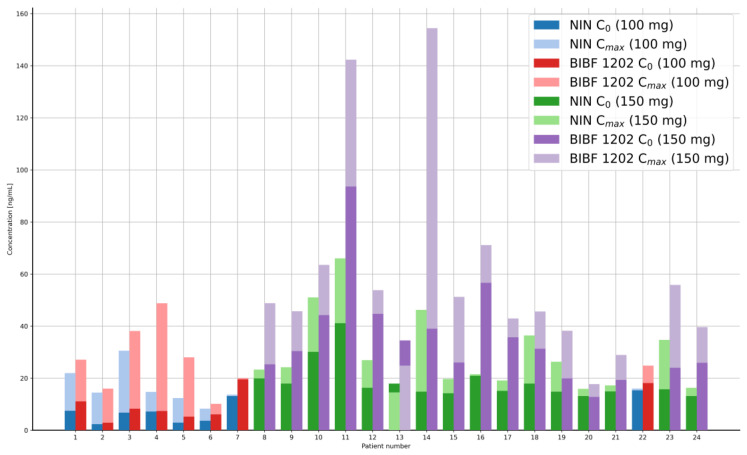
NIN and BIBF 1202 concentrations in plasma samples of patients treated with two doses: 100 and 150 mg of drugs.

The LC-MS/MS method developed and fully validated in this study was used to analyze the concentrations (C_0_ and C_max_) of nintedanib and its metabolite BIBF 1202 in human plasma. Twenty-four patients were treated with two doses of NIN (8 patients: 100 or 16 patients: 150 mg twice daily). In total, 48 samples were collected from patients at two time points: a morning dose at the trough level, and a dose 2–3 h (C_max_) after the morning dose administration. The concentrations of NIN and its metabolite were determined in the plasma of 24 patients (Male/Female 8:16, mean age 56.2, age range 35–75) with progressive pulmonary fibrosis associated with systemic sclerosis. The obtained results are presented in Table 7 and illustrated graphically (Figure 4).

## 4. Discussion

The method described in this article for determining NIN and BIBF 1202 in biological samples was developed and validated in accordance with EMA guidelines [16], allowing for its successful use in a clinical trial including 24 patients. The main challenge for the described assay was to reach the optimal separation between NIN and its main metabolite with high specificity, a low limit of detection, proper precision and accuracy, and an optimal time of analysis. This aim was achieved through the use of a highly sensitive XEVO-TQ-XS tandem quadrupole mass spectrometer (Waters) and an effective method for sample purification and the isolation of active substances.

There are a few procedures found in the literature regarding the development and validation of the LC-MS/MS method for the determination of NIN, but among them, only two are dedicated to the analysis of NIN in the human plasma of patients with progressive pulmonary fibrosis. Also, it was found that a few articles were published that conducted quantity analysis on rat biological fluids [13,14,15]. Veerman et al. created a very sensitive LC-MS/MS method for the determination of four molecule kinase inhibitors, including NIN, and 3 years later, a very ambitious clinical study was conducted using the developed analytical method for pharmacokinetic analysis in patients with pulmonary fibrosis [8,10]. Here, we would just like to note that quantifying the small-molecule kinase inhibitors applies in most instances in pharmacokinetic studies in patients with lung cancer [10]. However, previous studies have shown that kinase inhibitors also inhibit the expression of profibrotic factors that activate fibroblasts and stimulate the secretion of fibronectin and collagen. Despite the conclusion presented by the authors from Erasmus Medical Center that in the case of patients with pulmonary fibrosis, the implementation of TDM seems to be hardly justified [8], we recommended the correctness of such action, at least because of a large inter- and intra-patient variability in nintedanib pharmacokinetics, the side effects in the form of diarrhea, and the controlling of adherence.

Numerous experiments conducted during the method’s development allowed us to achieve the most optimal conditions for the isolation of active substances from biological samples as well as for the separation and detection conditions. A crucial element in optimizing the isolation of compounds from plasma is the volume of biological material used. Previous studies used various plasma volumes, ranging up to 1 mL [9], whereas in our method, the volume of biological material used was only 200 µL. This volume was sufficient to simultaneously determine both compounds with high sensitivity. In the described method, intedanib-d3 was used as an internal standard. NIN-d3 differs structurally from NIN only by three deuteriums, which guarantees nearly identical sample processing behavior and identical retention and recovery times during isolation from biological material. This ensures a reliable and reproducible quantitative determination of the analyte in biological material. Due to the minimal difference in the molecular structure, this internal standard was also suitable for determining BIBF 1202 concentration in plasma. Additionally, it is cheaper than the ^13^C,^2^H_3_-nintedanib proposed in the publication by Janssen et al. [11]. The use of analogs or compounds similar to IS [9,13,15] instead of labeled standards can lead to the insufficient correction of experimental variability during sample preparation and analysis, such as sample loss during transfer or additional filtration [9]. In the case of optimizing chromatographic conditions, it is very important to minimize retention time while maintaining high sensitivity and selectivity. In the presented method, the retention time of BIBF 1202 was 1.21 min, and that of NIN and IS was 1.35 min. This is possible through the use of a highly sensitive MS/MS detector that distinguishes compounds based on the *m*/*z* ratio, without the need to separate compounds on a chromatographic column. Compared to previous methods, the retention times were similar [9] or longer for NIN: 1.43 min [11], 5.0 min [10], and 5.98 min [12].

An additional advantage of this method is the simple composition of the mobile phase, which does not require complicated preparation, unlike previously published methods [9,10,11]. The same mobile phase composition was described by Lin et al. [13] and Xu et al. [15], with the difference that in the presented method, the elution was simplified from gradient to isocratic without compromising the sensitivity of the method. Methods for the simultaneous determination of NIN and BIBF 1202 in biological material are already known, but only in animals [13,15]. The method described in our publication was successfully applied to human plasma. A comparison of the selected methods is presented in Table 8.

The determination of NIN and BIBF 1202 concentrations was performed in forty-eight plasma samples collected from patients with PPF. The measured concentrations, C_0_ and C_max_ (approx. 3 h after dose administration), were all within the range of the calibration curve. Peak plasma concentrations of NIN typically occur 2–3 h after administration according to the PK study presented in the literature. The developed and validated method used to determine NIN and BIBF 1202 concentrations in biological samples is simple, rapid, and cost-effective.

The limitations of our study include the relatively small group of patients, which prevents the correlation of NIN and BIBF 1202 concentrations with clinical outcomes. Additionally, conducting TDM of NIN at the two time points, for ethical reasons, makes it impossible to compare our results with those of previous studies including multi-point pharmacokinetics profiles. The proposed methodology is intended for adult patients. With drug monitoring in the pediatric population in mind, the method should be refined. Lower drug doses are typically administered in the pediatric population, and, therefore, expected plasma concentrations will be correspondingly lower than in the adult population. This method should be improved by lowering the LLOQ value in the calibration curve. Furthermore, for bioethical reasons, the method should incorporate a smaller volume of biological material in the pediatric population.

## 5. Conclusions

We developed and validated a sensitive LC-MS/MS analytical method for the simultaneous quantification of nintedanib and its metabolite BIBF 1202 in human plasma. Further study will be necessary on a larger group of patients with PPF associated with systemic sclerosis in order to confirm the benefits of therapeutic drug monitoring in the case of therapy using kinase inhibitors. This could help with control adherence and the proper absorption of NIN. It could also be an element of correctly conducted pharmacotherapy.

## Figures and Tables

**Table 1:** MS instrument and analyte parameters.

General Parameters of Instrument			
**Parameter**	Value		
**Capillary voltage**	3.8 kV		
**Desolvation gas flow rate (N_2_)**	1000 L/h		
**Cone gas flow rate (N_2_)**	150 L/h		
**Ion source temperature**	150 °C		
**Desolvation gas temperature**	600 °C		
**Analyte-specific parameters**			
**Parameter**	NIN	BIBF 1202	IS
**MRM transition (*m*/*z*)**	540.27 → 113.00	526.27 → 112.95	543.29 → 116.06
**Cone voltage (v)**	75	43	62
**Collision energy (eV)**	24	20	24

**Table 2:** Results of the analysis of precision and accuracy of the developed ULPC-MS/MS method for the determination of NIN and BIBF 1202 in human plasma.

Nominal Concentration [ng/mL]	Compound	Run	Measured Concentration [ng/mL]	Accuracy [%]	Precision [%]
**2.00** **LLOQ**	**NIN**	Within-run	2.10 ± 0.06	102.2–107.3	2.96
		Between-run	2.00 ± 0.09	98.0–101.8	4.53
	**BIBF 1202**	Within-run	2.19 ± 0.12	104.3–114.2	5.51
		Between-run	2.04 ± 0.14	99.1–104.9	6.72
**5.00** **LQC_1_**	**NIN**	Within-run	4.82 ± 0.08	95.1–97.6	1.61
		Between-run	4.67 ± 0.12	92.5–94.5	2.66
	**BIBF 1202**	Within-run	4.78 ± 0.13	93.4–97.6	2.62
		Between-run	4.60 ± 0.15	90.8–93.2	3.23
**20.00** **LQC_2_**	**NIN**	Within-run	21.89 ± 0.29	108.2–110.6	1.32
		Between-run	21.75 ± 0.51	107.7–109.8	2.33
	**BIBF 1202**	Within-run	20.34 ± 0.92	97.9–105.5	4.55
		Between-run	20.88 ± 1.06	102.3–106.6	5.07
**100.00** **MQC**	**NIN**	Within-run	99.03 ± 1.44	97.8–100.2	1.46
		Between-run	99.34 ± 1.85	98.6–100.1	1.86
	**BIBF 1202**	Within-run	90.34 ± 1.77	88.9–91.8	1.96
		Between-run	94.08 ± 4.09	92.4–95.8	4.34
**170.00** **HQC**	**NIN**	Within-run	172.32 ± 1.46	100.7–102.1	0.85
		Between-run	169.74 ± 5.07	98.6–101.1	2.99
	**BIBF 1202**	Within-run	156.51 ± 2.44	90.9–93.2	1.56
		Between-run	160.32 ± 10.31	91.7–96.9	6.43

**Table 3:** The results of this study on the matrix effect; N—standard plasma; H—hemolyzed plasma; L—hyperlipidemic plasma.

Nominal Concentration [ng/mL]	Plasma Source	Compound	Measured Concentration [ng/mL]	Accuracy [%]	Precision [%]
**5.00**	**N1**	NIN	4.80 ± 0.08	94.7–97.4	1.71
		BIBF 1202	4.43 ± 0.18	85.6–91.5	4.10
	**N2**	NIN	4.75 ± 0.32	89.7–100.3	6.76
		BIBF 1202	4.54 ± 0.18	87.8–93.8	3.96
	**N3**	NIN	4.69 ± 0.10	92.0–95.4	2.19
		BIBF 1202	4.57 ± 0.16	88.8–94.2	3.55
	**N4**	NIN	4.61 ± 0.06	91.2–93.3	1.36
		BIBF 1202	4.82 ± 0.10	94.8–98.0	2.04
	**L**	NIN	4.52 ± 0.10	88.8–91.9	2.14
		BIBF 1202	4.39 ± 0.08	86.4–89.2	1.93
	**H**	NIN	4.54 ± 0.13	88.7–92.9	2.80
		BIBF 1202	4.38 ± 0.14	85.3–89.8	3.10
**170.00**	**N1**	NIN	177.22 ± 1.59	103.5–105.0	0.90
		BIBF 1202	164.22 ± 2.18	95.5–97.7	1.33
	**N2**	NIN	171.84 ± 2.18	100.0–102.1	1.27
		BIBF 1202	153.96 ± 4.38	88.4–92.7	2.84
	**N3**	NIN	173.70 ± 5.43	99.5–104.8	3.13
		BIBF 1202	157.18 ± 6.47	89.3–95.6	4.12
	**N4**	NIN	171.53 ± 8.43	96.8–105.0	4.91
		BIBF 1202	159.51 ± 5.59	91.1–96.5	3.50
	**L**	NIN	175.66 ± 9.17	98.9–107.8	5.22
		BIBF 1202	161.28 ± 8.57	90.7–99.0	5.31
	**H**	NIN	171.71 ± 3.18	99.5–102.5	1.85
		BIBF 1202	161.82 ± 8.41	91.1–99.3	5.20

**Table 4:** Results of the recovery.

Nominal Concentration [ng/mL]	Recovery [%]		
	NIN	BIBF 1202	IS
**5.00**	87.27	72.59	87.74
**100.00**	86.82	72.64	86.56
**170.00**	83.73	71.65	85.07

**Table 5:** Stability of samples containing NIN and BIBF 1202 in different conditions.

QC Samples of Analyzed Compounds NIN and BIBF 1202		Reference Samples [ng/mL]	Study Samples [ng/mL]	Stability [%]
**Short-term stability at ambient temperature** **(≤30 °C)** **(4 h for NIN and 2 h for BIBF 1202)**				
**LQC_1_**	**NIN**	4.60 ± 0.06	4.63 ± 0.12	100.7
	**BIBF 1202**	4.52 ± 0.13	4.55 ± 0.09	100.8
**HQC**	**NIN**	166.34 ± 7.28	168.58 ± 1.61	101.3
	**BIBF 1202**	149.78 ± 3.85	153.25 ± 10.51	102.3
**Autosampler stability at 10 ± 2 °C, after 45 h**				
**LQC_1_**	**NIN**	4.93 ± 0.09	4.75 ± 0.06	96.4
	**BIBF 1202**	4.54 ± 0.05	4.47 ± 0.15	98.5
**HQC**	**NIN**	172.52 ± 1.42	173.53 ± 1.63	100.6
	**BIBF 1202**	163.63 ± 1.26	161.90 ± 1.88	98.9
**Freeze–thaw stability after 3 cycles**				
**LQC_1_**	**NIN**	4.60 ± 0.06	4.35 ± 0.10	94.6
	**BIBF 1202**	4.77 ± 0.12	4.52 ± 0.12	94.7
**HQC**	**NIN**	166.34 ± 7.28	165.92 ± 1.20	99.7
	**BIBF 1202**	156.51 ± 2.44	165.72 ± 4.19	105.9

**Table 6:** Results of incurred sample reanalysis.

Analyte	Patient No.	Original Result [ng/mL]	Repeat Result [ng/mL]	% Difference [%]
**NIN**	1	10.81	12.55	14.6
	7	19.43	21.90	12.1
	6	7.64	9.22	19.0
	20	8.47	9.41	11.2
	5	6.12	7.13	15.1
**BIBF 1202**	3	33.30	39.52	17.0
	23	58.81	64.14	8.6
	9	35.11	42.26	18.4
	10	66.24	71.12	7.1
	16	70.91	75.00	5.6

**Table 7:** The results of NIN and BIBF 1202 plasma concentrations in patients treated with two doses: 100 or 150 mg of drug twice daily.

Dose * [mg]			Mean Concentration [ng/mL]	SD	RSD [%]
**100.0**	**NIN**	C_0_	7.33	4.71	64.26
	*n* = 8	C_max_	16.50	6.84	41.44
	**BIBF 1202**	C_0_	9.81	6.04	61.54
	*n* = 8	C_max_	26.61	12.29	46.19
**150.0**	**NIN**	C_0_	18.60	7.28	39.14
	*n* = 16	C_max_	28.70	14.66	51.09
	**BIBF 1202**	C_0_	35.20	19.11	54.28
	*n* = 16	C_max_	57.80	37.94	65.67

* dose given to patient twice daily; *n*: patient number.

**Table 8:** LC-MS/MS methods for determination of NIN in plasma.

Study	Plasma	Linear Range [ng/mL]	Isolation	IS	Mobile Phase	Elution	BIBF 1202 Determination	Comments
Presented method	human	2–200 for NIN 2–200 for BIBF 1202	PP	intedanib-d3	A: 0.1% HCOOH in water B: ACN	isocratic	YES	with clinical applicability
Veerman et al., 2021 [10]	human	5–100 for NIN	SPE	dasatinib-d8	A: H_2_O/HCOOH/ammonium formate (100:0.1:0.02, *v/v/v*) B: MeOH/HCOOH (100:0.1, *v/v*)	gradient	No	Needle wash: ACN/MeOH/2-propanol/H_2_O/HCOOH (25:25:25:25:0.1, *v/v/v/v/v*)
Janssen et al., 2019 [11]	human	10–200 for NIN	PP	^13^C,^2^H_3_-nintedanib	A: 10 mM ammonium bicarbonate (pH 10.5) in H_2_O B: 10 mM ammonium bicarbonate (pH 10.5) in MeOH/H_2_O (1:9, *v/v*)	gradient	No	autosampler vial with insert that contained 100 µL 10 mM ammonium bicarbonate in water *
Lin et al., 2016 [13]	rat	1–200 for NIN 0.5–100 for BIBF 1202	PP	diazepam	A: 0.1% HCOOH in water B: ACN	gradient	YES	rat plasma, without labeled internal standard
Darwish et al., 2016 [9]	human	2–150 for NIN	PP	cyklobenzaprine	A: 0.01 M ammonium formate (pH 4.2) B: ACN	isocratic	No	0.22 µm syringe filter **, without labeled internal standard, without clinical applicability
Xu et al., 2015 [15]	mouse	1–1000 for NIN 1–1000 for BIBF 1202	PP	carbamazepine	A: 0.1% HCOOH in water B: ACN	gradient	YES	mouse plasma, without labeled internal standard

PP—protein precipitation, SPE—Solid Phase Extraction; * 100 µL supernatant was added to an amber-colored autosampler vial with insert that contained 100 µL 10 mM ammonium bicarbonate in water; ** supernatants were filtered through a Millex-GP, 0.22 µm syringe filter.

## Data Availability

The raw data supporting the conclusions of this article will be made available by the authors on request.

## Abbreviations

The following abbreviations are used in this manuscript:

BS	Blank sample
BIBF 1202	Main metabolite of nintedanib acronym
EMA	European Medicines Agency
NIN-d3	Intedanib-d3, isotope-labeled internal standard
ISR	Incurred sample reanalysis
LLOQ	Lower limit of quantification
NIN	Nintedanib
PPF	Progression of pulmonary fibrosis
TDM	Therapeutic drug monitoring
LC-MS/MS	Liquid Chromatography–Tandem Mass Spectrometry
QC	Quality control samples
